# Motion as a source of environmental information: a fresh view on biological motion computation by insect brains

**DOI:** 10.3389/fncir.2014.00127

**Published:** 2014-10-28

**Authors:** Martin Egelhaaf, Roland Kern, Jens Peter Lindemann

**Affiliations:** Department of Neurobiology and Center of Excellence “Cognitive Interaction Technology” (CITEC), Bielefeld UniversityBielefeld, Germany

**Keywords:** optic flow, motion detection, spatial vision, insects, natural environments

## Abstract

Despite their miniature brains insects, such as flies, bees and wasps, are able to navigate by highly erobatic flight maneuvers in cluttered environments. They rely on spatial information that is contained in the retinal motion patterns induced on the eyes while moving around (“optic flow”) to accomplish their extraordinary performance. Thereby, they employ an active flight and gaze strategy that separates rapid saccade-like turns from translatory flight phases where the gaze direction is kept largely constant. This behavioral strategy facilitates the processing of environmental information, because information about the distance of the animal to objects in the environment is only contained in the optic flow generated by translatory motion. However, motion detectors as are widespread in biological systems do not represent veridically the velocity of the optic flow vectors, but also reflect textural information about the environment. This characteristic has often been regarded as a limitation of a biological motion detection mechanism. In contrast, we conclude from analyses challenging insect movement detectors with image flow as generated during translatory locomotion through cluttered natural environments that this mechanism represents the contours of nearby objects. Contrast borders are a main carrier of functionally relevant object information in artificial and natural sceneries. The motion detection system thus segregates in a computationally parsimonious way the environment into behaviorally relevant nearby objects and—in many behavioral contexts—less relevant distant structures. Hence, by making use of an active flight and gaze strategy, insects are capable of performing extraordinarily well even with a computationally simple motion detection mechanism.

## Environmental information as a basis for visually guided orientation

A key function of vision is to extract behaviorally relevant information about the outside world from the activity patterns evoked in the retina. Especially fast locomotion requires information about the spatial layout of the environment to allow for meaningful behavioral decisions. Spatial information can be obtained from the relative movements of the retinal image, the optic flow patterns that are generated on the eyes during locomotion.

Visual motion information is not only generated on the eyes when a moving object crosses the visual field, but also all the time while the animal moves around in the environment. Despite this ongoing movement on the retina, we usually perceive the outside world as static. Nevertheless, the retinal motion information is conventionally thought to be important to signal self-motion. One particular type of self-motion has been studied intensively, especially in tethered animals: confronted with a rotating environment, most animals generate eye- or body-movements following this rotation. These rotational responses of the eyes and/or the body to visual motion were monitored and interpreted to compensate for deviations from an intended course of locomotion or an intended gaze direction. In this context the retinal motion is regarded as a disturbance that needs to be compensated (reviews: Götz, 1972; Taylor and Krapp, 2008; Borst, 2014). Although this view may be correct in many behavioral situations, it misses one important point: retinal image motion induced by self-motion of the animal is not just a nuisance, but may also be a highly relevant source of environmental information. In particular, fast flying animals, such as many insects, heavily rely on environmental information derived from optic flow, for instance, to avoid collisions with obstacles, to find a landing site and control landing maneuvers or when learning the landmark constellation around a goal and when later navigating towards this previously learnt site. However, also sitting animals may induce specific body, head, and eye movements for estimating distances to objects in their environment (for review see Collett and Zeil, 1996; Kral, 2003; Srinivasan, 2011; Egelhaaf et al., 2012; Zeil, 2012).

The working hypothesis of much of our recent research on insects, such as flies and bees (Egelhaaf et al., 2012) and, thus, the assumption underlying this article is that the output of the motion vision system combines two highly relevant cues of environmental information: nearness and contrast borders. As a consequence it segments the time-dependent retinal images into potentially relevant nearby structures and—in many behavioral contexts–potentially less relevant distant objects. On the one hand, we will argue that all this is likely to be accomplished by simple computational principles that have been conceptually lumped into a well-known and well-established computational model, i.e., the correlation-type movement detector (often also termed Hassenstein-Reichardt detector or elementary motion detector, EMD; Reichardt, 1961; Borst and Egelhaaf, 1989; Egelhaaf and Borst, 1993; Borst, 2000). On the other hand, we will sketch the current knowledge about how local motion information is further processed to guide orientation behavior.

## Insect motion detection reflects the properties of the environment in addition to velocity

The correlation-type motion detection scheme has been derived originally as a computational model on the basis of behavioral and electrophysiological experiments on insects (Reichardt, 1961; Egelhaaf and Borst, 1993; Borst et al., 2003; Borst, 2004; Lindemann et al., 2005; Straw et al., 2008; Brinkworth and O’Carroll, 2010; Meyer et al., 2011). Only recently, the computational principles are being decomposed on the circuit level. The neural networks and synaptic interactions underlying motion detection are investigated mainly in the fruitfly *Drosophila* by employing the sophisticated repertoire of novel genetic tools (e.g., Freifeld et al., 2013; Joesch et al., 2013; Maisak et al., 2013; Reiser and Dickinson, 2013; Silies et al., 2013; Tuthill et al., 2013; Behnia et al., 2014; Hopp et al., 2014; Mauss et al., 2014; Meier et al., 2014; Strother et al., 2014). Since we are focusing here especially on the overall output of the motion detection system, rather than on the cellular details of its internal structure, our considerations are mainly based on model analyses of EMDs. Variants of this computational model can account for many features of motion detection, as they manifest themselves in the activity of output cells of the motion vision pathway and even in the behavioral performance of the entire animal.

**Figure 1:**
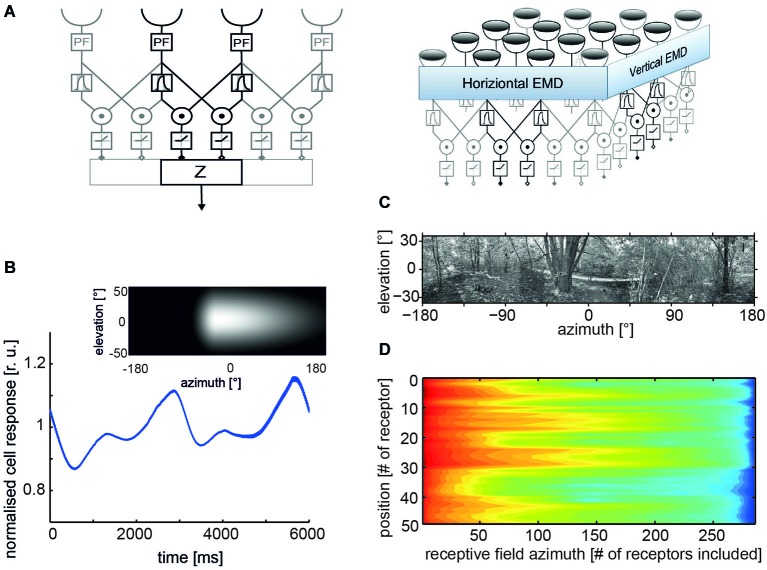
**Properties of correlation-type elementary movement detectors (EMDs). (A)** Structure of a basic variant of three neighboring movement detectors including peripheral filtering (PF) in the input lines; signals from each receptor are delayed via the phase delay of a temporal first-order low-pass filter, multiplied and half-wave rectified; spatial pooling of signals in accomplished by the output element Z (left); a two-dimensional EMD array consisting of EMDs most sensitive to horizontal and vertical motion, respectively (right). **(B)** Time course of pattern-dependent response modulations of model cell that pools the responses of an array of EMDs with horizontal preferred direction. The spatial sensitivity distribution of the model cell is given by the weight field shown in the inset. The brighter the gray level the larger the local weight of the corresponding EMDs and, thus, the spatial sensitivity. The frontal equatorial viewing direction is at 0° azimuth and 0° elevation. The model cell was stimulated by horizontal constant velocity motion of the panoramic high dynamic range image shown in **(C)**. **(D)** Logarithmic color coded standard deviation of the mean pattern-dependent modulation for one-dimensional receptive fields differing in vertical receptor position and azimuthal receptive field size (# of receptors included horizontally). The pattern-dependent modulation amplitude decreases with horizontal receptive field extent. They depend on the contrast distribution of the input image, as can be seen, when comparing pattern-dependent modulation amplitudes corresponding to the different elevations of the input image. (Data from Meyer et al., 2011).

In its simplest form, an EMD is composed of two mirror-symmetrical subunits (Figure 1A). In each subunit, the signals of adjacent light-sensitive cells receiving the filtered brightness signals from neighboring points in visual space are multiplied after one of them has been delayed. The final detector response is obtained by subtracting the outputs of two such subunits with opposite preferred directions, thereby considerably enhancing the direction selectivity of the motion detection circuit. Each motion detector reacts with a positive signal to motion in a given direction and with a negative signal to motion in the opposite direction (reviews: Reichardt, 1961; Borst and Egelhaaf, 1989, 1993). Various elaborations of this basic motion detection scheme have been proposed to account for the responses of insect motion-sensitive neurons under a wide range of stimulus conditions including even natural optic flow as experienced under free-flight conditions (e.g., Borst et al., 2003; Lindemann et al., 2005; Shoemaker et al., 2005; Brinkworth et al., 2009; Hennig et al., 2011; Hennig and Egelhaaf, 2012).

As a consequence of their computational structure, EMDs and their counterparts in the insect brain have a number of peculiar features that deviate in many respects from those of veridical velocity sensors. Therefore, they often have been interpreted as the consequence of a simple, but somehow deficient computational mechanism. The most relevant of these features are:

- *Ambiguous velocity dependence*: EMDs do not operate like speedometers: their mean responses increase with increasing velocity, reach a maximum, and then decrease again. The location of the velocity optimum depends on the spatial frequency composition of the stimulus pattern (Egelhaaf and Borst, 1993), but also on stimulus history and the behavioral state of the animal (Maddess and Laughlin, 1985; Egelhaaf and Borst, 1989; Warzecha et al., 1999; Kurtz et al., 2009; Chiappe et al., 2010; Longden and Krapp, 2010; Maimon et al., 2010; Rosner et al., 2010; Jung et al., 2011; Longden et al., 2014). At least the pattern dependence of velocity tuning is reduced if the stimulus pattern consists of a broad range of spatial frequencies, as is characteristic of natural scenes (Dror et al., 2001; Straw et al., 2008).
- *Contrast dependence*: the response of EMDs, at least in their most basic form, depends strongly on contrast, being a consequence of the multiplicative interaction between the two EMD input lines (Borst and Egelhaaf, 1989; Egelhaaf and Borst, 1993). This contrast dependence can be reduced to some extent by saturation nonlinearities or more elaborate contrast normalization measures (Egelhaaf and Borst, 1989; Shoemaker et al., 2005; Babies et al., 2011).
- *Pattern dependence of time-dependent responses*: owing to the small receptive fields of EMDs, their responses are temporally modulated even during pattern motion at a constant velocity. The modulations are a consequence of the texture of the environment. Since neighboring EMDs receive, at a given time, their inputs from different parts of the environment, their output signals modulate with a different time course. As a consequence, spatial pooling over EMDs reduces mainly those pattern-dependent response modulations that originate from the high spatial frequencies of the stimulus pattern (Figure 1B). The pattern-dependent response modulations decrease with increasing the spatial pooling range (Figures 1C,D; Egelhaaf et al., 1989; Single and Borst, 1998; Meyer et al., 2011; O’Carroll et al., 2011; Schwegmann et al., 2014).
- *Motion adaptation*: the responses of motion vision systems were found to depend on stimulus history and to be adjusted by a variety of mechanisms to the prevalent stimulus conditions (reviews: Clifford and Ibbotson, 2002; Egelhaaf, 2006; Kurtz, 2012). These processes are usually regarded as adaptive, although their functional significance is still not entirely clear. Several non-exclusive functional roles have been proposed, such as adjusting the dynamic range of motion sensitivity to the prevailing stimulus dynamics (Brenner et al., 2000; Fairhall et al., 2001), saving energy by adjusting the neural response amplitudes without affecting the overall information that is conveyed (Heitwerth et al., 2005), and increasing the sensitivity to temporal discontinuities in the retinal input (Maddess and Laughlin, 1985; Liang et al., 2008, 2011; Kurtz et al., 2009).

These response features of EMDs make their responses ambiguous with respect to a representation of the retinal velocity. Because these ambiguities, especially the contrast- and texture-dependent response modulations, deteriorate the quality of representing pattern velocity, they have often been discussed as “pattern noise” (Dror et al., 2001; Shoemaker et al., 2005; Rajesh et al., 2006; O’Carroll et al., 2011) and, thus, as a limitation of the biological motion detection mechanism. Here we want to take an alternative stance by proposing that these pattern-dependent modulations of the movement detector output do not reflect noise in the context of velocity coding. Rather, they can be interpreted as being relevant from a functional point of view, as they reflect potentially useful information about the environment and, thus, may be relevant for visually guided orientation behavior (Meyer et al., 2011; O’Carroll et al., 2011; Hennig and Egelhaaf, 2012; Schwegmann et al., 2014; Ullrich et al., 2014b).

## Enhancing the overall power of insect brains: reducing computational load by active vision strategy

It is indispensable that the animal is active and moves to be able to use the environmental information provided by EMDs. This is because movement detectors do not respond in a stationary world if the animal is also stationary. However, not every type of self-motion is equally suitable for the brain to extract useful information about the environment from the image flow and, thus, from the EMD responses. Especially, if spatial information is concerned only the optic flow component generated by translational self-motion is useful. During pure translational self-motion the retinal images of objects close to the observer move faster than those of more distant ones. More specifically, for a given translation velocity, retinal image velocity evoked by an environmental object at a given viewing angle increases linearly with its nearness, i.e., the inverse of its distance. However, the retinal velocity of an object even at a given distance also depends on its viewing angle relative to the direction of motion: the optic flow vectors are maximal at 90° relative to the direction of motion and decrease according to a sine function from here towards the direction of self-motion, where they are zero. Hence, at this singular point, i.e., the direction in which the agent is heading, it is not possible to obtain nearness information. The geometrical situation differs much for pure rotational self-movements of the agent. Then the retinal image displacements are independent of the distance to objects in the environment (Koenderink, 1986).

If locomotion is characterized by an arbitrary combination of translation and rotation, the optic flow field is more complex, and information about the spatial structure of the environment cannot readily be derived. Nevertheless, a segregation of the optic flow into its rotational and translational components can, at least in principle, be accomplished computationally for most realistic situations (Longuet-Higgins and Prazdny, 1980; Prazdny, 1980; Dahmen et al., 2000). However, such a computational strategy is demanding, and it is not clear whether it can be pursued by a nervous system. Several insect species with their tiny brains appear to employ other computationally much more parsimonious strategies.

**Figure 2:**
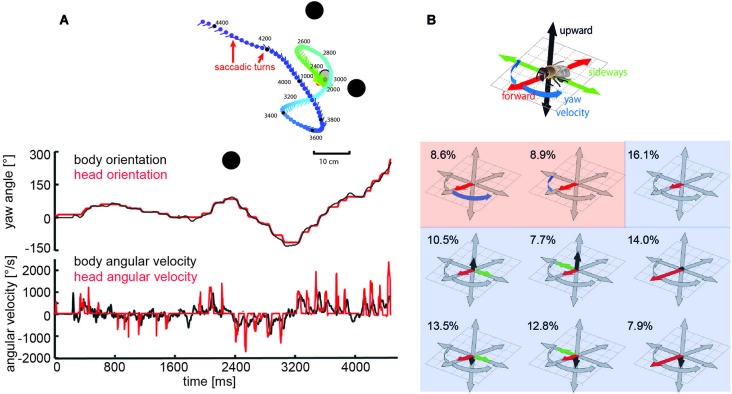
**Saccadic flight strategy and variability of translational self-motion**. Saccadic flight and gaze strategy of free-flying bumblebees and honeybees. **(A)** Inset: Trajectory of a typical learning flight of a bumblebee as seen from above during a navigational task involving landmarks (black objects). Each line indicates a point in space and the corresponding viewing direction of the bee’s head each 20 ms. The color code indicates time (given in ms after the start of the learning flight at the goal). Upper diagram: Angular orientation of longitudinal axis of body (black line) and head (red line) of a sample flight trajectory of a bumblebee during a learning flight after departing from a visually inconspicuous feeder surrounded by three landmarks. Note that step-like, i.e., saccadic direction changes are more pronounced for the head than for the body. Bottom diagram: Angular yaw velocity of body (black line) and head (red line) of the same flight (Boeddeker et al., submitted; Data from Mertes et al., 2014). **(B)** Translational and rotational prototypical movements of honeybees during local landmark navigation. Flight sequences while the bee was searching for a visually inconspicuous feeder located between three cylindrical landmarks can be decomposed into nine prototypical movements using clustering algorithms in order to reduce the behavioral complexity. Each prototype is depicted in a coordinate system as explained by the inset. The length of each arrow determines the value of the corresponding velocity component. Percentage values provide the relative occurrence of each prototype. More than 80% of flight-time corresponds to a varied set of translational prototypical movements (light blue background) and less than 20% has significantly non-zero rotational velocity corresponding to the saccades (light red background) (Data from Braun et al., 2012).

**Figure 3:**
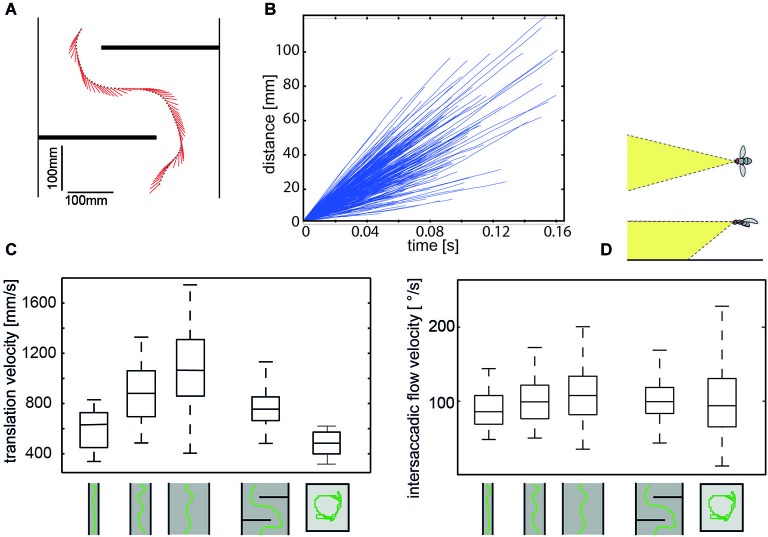
**Regulation of intersaccadic retinal velocity by context-dependent control of flight speed**. **(A)** Sample flight trajectory of a blowfly as seen from above negotiating an obstacle in a flight tunnel (only middle section shown); the position of the fly (black dot) and the projection of the orientation of the body long axis in the horizontal plane (red line) are given every 10 ms. **(B)** Distance flown within individual intersaccades as a function of the time that is needed. Shown are incremental distance vs. time plots for 320 intersaccadic intervals obtained from 10 spontaneous flights in a cubic box (see right pictogram in **(C)** (Flight trajectories provided by van Hateren and Schilstra, 1999). **(C)** Boxplot of the translational velocity in flight tunnels of different widths, in a flight arena with two obstacles and in a cubic flight arena (sketched below data). Translation velocity strongly depends on the geometry of the flight arena (Data from Kern et al., 2012). **(D)** Boxplot of the retinal image velocities within intersaccadic intervals experienced in the fronto-ventral visual field (see inset above boxplot) in the different flight arenas. In this area of the visual field, the intersaccadic retinal velocities are kept roughly constant by regulating the translation velocity according to clearance with respect to environmental structures. The upper and lower margins of the boxes in **(C)** and **(D)** indicate the 75th and 25th percentiles, and the whiskers the data range (Data from Kern et al., 2012).

Specific combinations of rotatory and translatory self-motion may generate an optic flow pattern that contains useful spatial information. For instance, when the animal circles around a pivot point while fixating it, the retinal images of objects before and behind the pivot point move in opposite directions and, thus, provide distance information relative to the pivot point, rather than to the moving observer (Collett and Zeil, 1996; Zeil et al., 1996). Other insects generate pure translational self-motion to obtain distance information relative to the animal. For instance, mantids, dragonflies, and locusts, perform lateral body and head translations and employ the resulting optic flow for gaining distance information, when sitting in ambush to catch a prey or preparing for a jump (Collett, 1978; Sobel, 1990; Collett and Paterson, 1991; Kral and Poteser, 1997; Olberg et al., 2005). During flight, flies, wasps and bees reveal a distinctive behavior that is characterized by sequences of rapid saccade-like turns of body and head interspersed with virtually pure translational, i.e., straight flight phases (Schilstra and Van Hateren, 1999; van Hateren and Schilstra, 1999; Mronz and Lehmann, 2008; Boeddeker et al., 2010, submitted; Braun et al., 2010, 2012; Geurten et al., 2010; Kern et al., 2012; van Breugel and Dickinson, 2012; Zeil, 2012). Saccadic gaze changes have a rather uniform time course and are shorter than 100 ms. Angular velocities of up to several thousand °/s can occur during saccades (Figures 2A, 3A). Rotational movements associated with body saccades are shortened for the visual system by coordinated head movements and roll rotations performed for steering purposes during sideways translations, are compensated by counter-directed head movements. As a consequence, the animal’s gaze direction is kept virtually constant during intersaccades (Schilstra and Van Hateren, 1999; van Hateren and Schilstra, 1999; Boeddeker and Hemmi, 2010; Boeddeker et al., 2010, submitted; Braun et al., 2010, 2012; Geurten et al., 2010, 2012). Hence, turns that are essential to reach behavioral goals are minimized in duration and separated from translational flight phases in which the direction of gaze is kept largely constant. This peculiar time structure of insect flight facilitates the processing of distance information from the translational intersaccadic optic flow. With regard to gathering information about the outside world, it is highly relevant from a functional perspective that the intersaccadic translational motion phases last for more than 80% of the entire flight time (van Hateren and Schilstra, 1999; Boeddeker and Hemmi, 2010; Boeddeker et al., 2010; Braun et al., 2012; van Breugel and Dickinson, 2012). Still, the individual intersaccadic time intervals are short and usually last for only some ten milliseconds; they are only rarely longer than 100 to 200 ms in blowflies, for example (Kern et al., 2012). This characteristic dynamic feature of the active flight and gaze strategy of insects, thus, constrains considerably the timescales on which spatial information can be extracted from the optic flow patterns during flight, a fact the underlying neuronal mechanisms have to cope with (Egelhaaf et al., 2012).

Although the translational intersaccadic flight phases are diverse with regard to the direction and velocity of motion they appear to be adjusted to the respective behavioral context (Figure 2B; Braun et al., 2010, 2012; Dittmar et al., 2010; Geurten et al., 2010). This is especially true for the overall velocity of translational self-motion, although it does not change much during individual intersaccadic intervals (Figure 3B; Schilstra and Van Hateren, 1999; van Hateren and Schilstra, 1999; Boeddeker et al., 2010; Kern et al., 2012). For instance, insects tend to decelerate when their flight path is obstructed, and flight speed is thought to be controlled by optic flow generated during flight (David, 1979, 1982; Farina et al., 1995; Srinivasan et al., 1996; Kern and Varjú, 1998; Baird et al., 2005, 2006, 2010; Frye and Dickinson, 2007; Fry et al., 2009; Dyhr and Higgins, 2010; Straw et al., 2010; Kern et al., 2012). Thereby, they appear to regulate their intersaccadic translational flight velocity to keep the retinal velocities in the frontolateral visual field largely constant at a “preset” level (Baird et al., 2010; Portelli et al., 2011; Kern et al., 2012). This level appears to lie within the part of the operating range of the motion detection system where the response amplitude still increases with increasing retinal velocity (Figure 3; See Section Insect Motion Detection Reflects the Properties of the Environment in Addition to Velocity). These features are likely to be of functional significance from the perspective of spatial vision, because they help to reduce the ambiguities in extracting nearness information from the EMD outputs that represent the optic flow in the visual system. On the other hand, since insects may adjust their translational velocity to the behavioral context (see above, but also Srinivasan et al., 2000), no absolute nearness cues can be obtained by any mechanism extracting spatial information from optic flow: this is because a given retinal velocity and, thus, response level of a motion detection system may be obtained for different combinations of translation velocity and nearness. Hence, nearness information can be extracted only in relative terms, unless translation velocity is known. This implies that translation velocity should be kept constant, if from the response modulations of EMDs (See Section Insect Motion Detection Reflects the Properties of the Environment in Addition to Velocity) nearness information needs to be determined. If also the translation velocity varies, the resulting response modulations are ambiguous with regard to their origin: they could be a consequence of either changes in self-motion or the spatial structure of the surroundings.

## Representation of cluttered environments by arrays of motion detectors

Insects provide the basis for representing computationally efficient environmental information from the optic flow generated during the intersaccadic intervals of largely translational self-motion. However, optic flow information is not explicitly given at the retinal input. Rather, it needs to be computed from the spatiotemporal brightness fluctuations that are sensed by the array of photoreceptors of the retina. This is accomplished by local neural circuits residing in the visual neuropils. As explained in Section Insect Motion Detection Reflects the Properties of the Environment in Addition to Velocity the overall performance of these circuits can be lumped together and explained by variants of the correlation-type EMD. Despite the detailed knowledge at the cellular and computational level, the functional significance of the information provided by these movement detectors has not been clearly unraveled yet. Since EMDs are sensitive to velocity, they may exploit the different speeds of objects at different nearnesses during translational self-motion and, thus, may represent information about the depth structure of the environment. However, EMDs are also sensitive to textural features of the environment (See Section Insect Motion Detection Reflects the Properties of the Environment in Addition to Velocity). Is this pattern dependence of the EMD output just an unwanted by-product of a simple computational mechanism, or could it have any functional significance?

**Figure 4:**
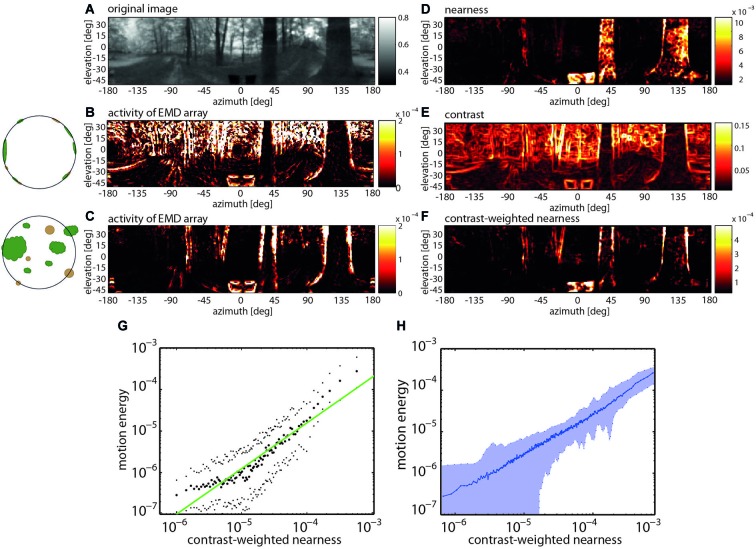
**Representation of nearby contours by EMD arrays during translatory self-motion. (A)** Panoramic input image with brightness adjusted to the spectral sensitivity of the motion detection system. **(B)** Activity profile of EMD array after equalizing the depth structure of the environment (see inset). **(C)** Activity profile of EMD array in response to translatory motion in environment with natural depth structure. **(D)** Nearness of environmental structures. **(E)** Local contrast of environmental structures. **(F)** Contrast-weighted nearness of environmental structures. **(G)** Relation between motion energy and the contrast-weighted nearness plotted in a double logarithmic way for the center of the track of the forest scenery shown in **(A)**. **(H)** Relation between motion energy and the contrast-weighted nearness for 37 full-depth motion sequences recorded in a wide range of different types of natural environments (Data from Schwegmann et al., 2014).

Recent model simulations of arrays of EMDs provided evidence that their pattern dependence may make sense from a functional perspective during translatory self-motion in cluttered natural environments. Although several experimental and modeling studies probed the insect motion vision system already before with moving natural images, they only employed image sequences that did not contain any depth structure and, thus, differed much from what an animal experiences in natural environments (Straw et al., 2008; Wiederman et al., 2008; Brinkworth et al., 2009; Barnett et al., 2010; Meyer et al., 2011; O’Carroll et al., 2011). The potential significance of the combined velocity and pattern dependence of correlation-type EMDs became obvious by comparing the activity profiles of EMD arrays induced by image sequences that were obtained from constant-velocity translational movements through a variety of cluttered natural environments containing the full depth information and after the depth structure of the environment was removed. For both types of situations, sample activity profiles of EMD arrays are shown in Figure 4. They differ much, because without depth structure all environmental objects move at the same velocity and, thus, lead to responses irrespective of their distance. It is obvious that the activity profile evoked by motion through the environment with its natural depth structure preserved is most similar not to the nearness map *per se*, but to the contrast-weighted nearness map, which is the nearness multiplied by the contrast. However, the activity profile evoked by the artificially depth-removed image sequences matches best the contrast map (Schwegmann et al., 2014). This exemplary finding is corroborated by correlation analysis based on translatory motion through several different natural environments (Figure 4).

Hence, EMD arrays do not respond best to the retinal velocity *per se* and, thus, to the nearness of environmental structures, but to the contrast-weighted nearness. This means that, during translational self-motion in natural environments, the arrays of EMDs represent to a large degree the nearness of high-contrast contours of objects. This conclusion holds true as long as the translational velocity varies only little and, thus, does not induce time-dependent response changes on its own (See Section Enhancing the Overall Power of Insect Brains: Reducing Computational Load by Active Vision Strategy). As mentioned above, this condition is met to a large extent for the short time of most intersaccadic intervals (Figure 3B; Schilstra and Van Hateren, 1999; van Hateren and Schilstra, 1999; Kern et al., 2012). By representing the contours of nearby objects, the distinctive feature of EMDs to jointly represent contrast and nearness information may make perfect sense from a functional point of view. Cluttered spatial sceneries are segmented in this way, without much computational expenditure, into nearby and distant objects. This finding underlines the notion that the mechanism of motion detection has been tweaked by evolution to allow the tiny brains of insects to gather behaviorally relevant information in a computationally efficient way.

However, motion measurements cannot be made instantaneously. As is reflected by the time constants that are an integral constituent of any motion detection mechanism including correlation-type EMDs, it may take some time until reliable motion information and, thus, spatial cues can be extracted from their responses. This may be a challenge as the uninterrupted translational movement phases during intersaccadic intervals are short, ranging between 30 ms up to little more than 100 ms (Figure 3B). It takes few milliseconds after a change from a saccadic rotation to an intersaccadic translational movement for the EMD response to reach a kind of steady-state level. This finding indicates that the initial part of a translational sequence cannot be used by the animal for a reliable estimation of nearness information from the EMD responses (Schwegmann et al., 2014). Even under such constraints the duration of most intersaccadic intervals appears to be long enough to allow for extracting spatial information from the optic flow patterns on the eyes.

In conclusion, during constant-velocity translatory locomotion the largest responses of the motion detection system are induced by contrast borders of nearby objects. Hence, it appears to be of functional significance that insects, such as flies and bees move essentially straight for more than 80% of their flight time and change their direction by interspersed saccadic turns of variable amplitude (Figure 2B). Since translation velocity does not change much during intersaccadic intervals, the output of the motion detection system during individual intersaccadic intervals highlights contrast borders of nearby objects. Thus, what has been conceived often to be a limitation of the insect motion detection system may turn out to be a means that allows—in combination with the active flight and gaze strategy—to parse the environment into near and far and, at the same time, enhance the representation of object borders in a computationally extremely parsimonious way. By combining contrast edge information and motion-based segmentation of the scene in a single representation, the insect vision pathway may reflect an elegant and computationally parsimonious mechanism for cue integration.

In computer vision optic flow is also used for segmentation purposes as well as for solving other spatial vision tasks, such as the recovery of the shape and relative depth of three-dimensional surface structures or the determination of the time-to-collision to an obstacle and the position of the focus of expansion to detect the heading direction (Beauchemin and Barron, 1995; Zappella et al., 2008). Since quite some time, a variety of approaches to optic flow computation has been proposed and applied to robotic applications. These algorithms are based on different assumptions on image motion and operate on different image representations, e.g., directly on the gray level values or the edges in the image sequences (Beauchemin and Barron, 1995; Fleet and Weiss, 2005). In contrast to EMDs that provide jointly information about motion and contrast edges during translatory motion, these technical optic flow approaches have in common that they attempt to estimate the optic flow field veridically, i.e., the flow vectors (up to a scaling factor) according to their velocity in the image plane. If applied to natural image sequence this, however, proofed to be possible to only some extent and erroneous velocity estimates are a common result depending on the pattern properties of the sceneries (Barron et al., 1994; McCarthy and Barnes, 2004). To what extent segmentation algorithms which compute segment borders from discontinuities in a dense field of optic flow estimates as provided by the various computer vision algorithms (Zappella et al., 2008) may be also applicable for computing segmentations based on a motion image computed by EMDs remains to be tested.

## Exploitation of environmental information from motion detectors by downstream mechanisms

Is the environmental information provided by the insect motion detection system during the translational phases of intersaccadic intervals really used by downstream processes in the nervous system and does it eventually play a role in controlling orientation behavior? Answers to this question can only be tentative so far, although it is suggested by two lines of evidence that the EMD-based environmental information might be functionally relevant. On the one hand, detailed knowledge is available of the computational properties of one neural pathway processing the information provided by the arrays of local motion detectors. On the other hand, behavioral studies and current modeling attempts suggest that the motion-based information about the environment may well be exploited for solving behavioral tasks such as collision avoidance and landmark navigation. Both aspects will be dealt with briefly in the following.

### Consequences of spatial integration

The output of the local motion sensitive elements in insects are spatially pooled to a varying degree in one neural pathway depending on the computational tasks that are being solved (Hausen, 1981; Krapp, 2000; Borst and Haag, 2002; Egelhaaf, 2006; Borst et al., 2010). However, spatial pooling inevitably reduces the precision with which a moving stimulus can be localized. Although this might appear, at least at first sight, to be a disadvantage, this is not necessarily the case. The determination of self-motion of the animal is one obvious task of motion vision systems. In this case, the retinal motion should not be localized, but rather only few output variables, i.e., of its translational as well as rotational velocities, are to be computed from the global optic flow. Information about self-motion is thought to be relevant for solving tasks such as, for instance, attitude control during flight, the compensation of involuntary disturbances by corrective steering maneuvers or the determination of the direction of heading (Dahmen et al., 2000; Lappe, 2000; Vaina et al., 2004; Taylor and Krapp, 2008; Egelhaaf et al., 2012). Accordingly, spatial pooling of local motion information over relatively large parts of the visual system as is done by wide-field cells (LWCs) in the lobula complex of insects enhances the specificity of the system for different types of self-motion (Hausen, 1981; Krapp et al., 1998, 2001; Franz and Krapp, 2000; Horstmann et al., 2000; Dror et al., 2001; Karmeier et al., 2003; Franz et al., 2004; Wertz et al., 2009).

**Figure 5:**
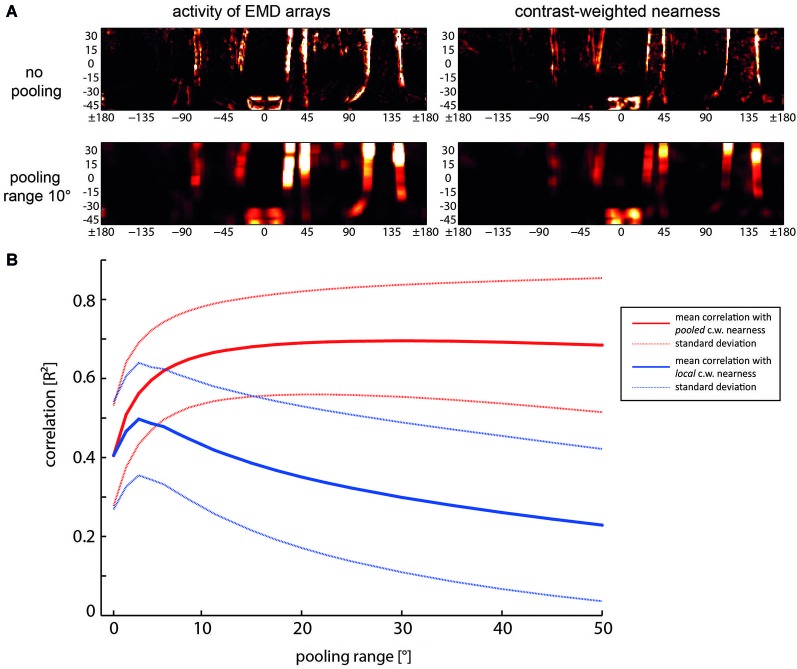
**Relationship between spatial pooling of local motion information, the reliability of representing nearby contours and their localizability. (A)** Examples of activity distribution of EMD arrays (left) and contrast-weighted nearness map (right) for no pooling (the upper row) and for a pooling range of 10°, i.e., spatially integrating the output a square array 8 × 8 neighboring EMDs (bottom row). **(B)** Mean correlation (solid lines) and standard deviations (dashed lines) as a function of pooling range. Red lines: Correlation of the pooled motion energy profile with the pooled contrast-weighted (c.w.) nearness map. Blue line: Correlation of the pooled motion energy profile with local non-pooled contast-weighted nearness map, indicating the reduction of localizability with increasing pooling range (Data from Schwegmann et al., 2014).

In contrast, if information about the spatial layout of the environment is required, it might be relevant to localize objects together with their nearness to the animal. Then spatial pooling over only a relatively small spatial area will be acceptable. Integration of the outputs of neighboring EMDs was found to increase considerably the reliability with which the boundaries of nearby objects are represented in the activity profile of EMDs; pooling of the direct and second neighbors is already sufficient. Increasing the pooling area further does not increase the contrast-weighted nearness information significantly, but reduces the localizability of environmental features to a spatial range as given by the receptive field size of the pooling neuron (Figure 5; Schwegmann et al., 2014). Spatial pooling across larger areas of the visual field provides only information about the averaged spatial information within the pooling areas during translational self-motion without being able to localize environmental features within this area of the visual field.

**Figure 6:**
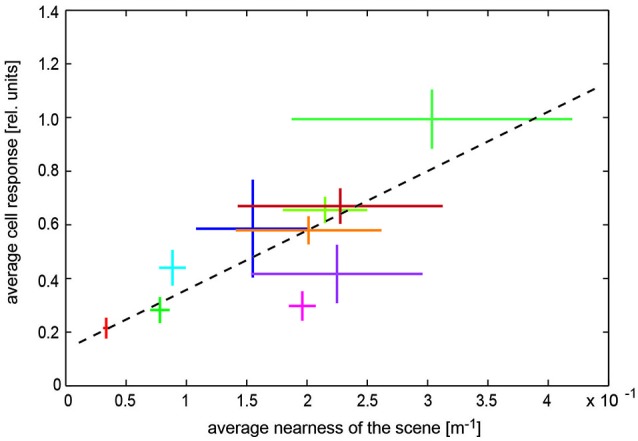
**Representation of spatial information by visual wide-field neurons**. Dependence of blowfly LWC with large receptive field (H1 neuron) on overall nearness during translatory self-motion in various cluttered natural environments. Data obtained in different environments are indicated by different colors. Horizontal bars: Standard deviation of the “time-dependent nearness” during the translation sequence within a given scenery, indicating the difference in the spatial structure of the different environments. Vertical bars: Standard deviation of response modulations obtained during the translation sequence in a given scenery. Corresponding mean values are given by the crossing of the horizontal and vertical bars. Regression line (black dashed line) illustrating the relation between nearness values and cell responses (Data from Ullrich et al., 2014a).

Experimentally most information about how the spatial layout of the environment might be represented by the visual motion pathway during translational self-motion is available from recent experiments on LWCs, those neurons that have usually been conceived as sensors for self-motion estimation because of their relatively large receptive fields (see above). However, individual LWCs are far from being ideal for self-motion estimation as their receptive fields are spatially clearly restricted and show distinct spatial sensitivity peaks. Accordingly, they show pronounced response modulations even during constant-velocity motion resulting from textural features of the environment (Meyer et al., 2011; O’Carroll et al., 2011; Ullrich et al., 2014b). In addition, the responses of LWCs provide information about the spatial layout of the environment—at least on a coarse spatial scale, but even on the short timescale of intersaccadic intervals: the intersaccadic response amplitudes evoked by ego-perspective movies were found to depend on the distance to the walls of the flight arena in which the corresponding behavioral experiments were performed or on objects that were inserted close to the flight trajectory (Boeddeker et al., 2005; Kern et al., 2005; Karmeier et al., 2006; Liang et al., 2008, 2012; Hennig and Egelhaaf, 2012). Moreover, LWC responses are found to reflect the overall depth structure of different natural environments (Figure 6; Ullrich et al., 2014a). Recently, it could even been shown that the intersaccadic responses of bee LWCs to visual stimuli as experienced during navigation flights in the vicinity of a goal strongly depend on the spatial layout of the environment. The spatial landmark constellation that guides the bees to their goal leads to a characteristic time-dependent response profile in LWCs during the intersaccadic intervals of navigation flights (Mertes et al., 2014).

What is the range within which spatial information is represented on the basis of motion information? Under spatially constrained conditions with the flies flying at translational velocities of only slightly more than 0.5 m/s, the spatial range within which significant distance dependent intersaccadic responses are evoked amounts to approximately two meters (Kern et al., 2005; Liang et al., 2012). Since a given retinal velocity is determined in a reciprocal way by distance and velocity of self-motion, respectively, the spatial range that is represented by LWCs can be expected to increase with increasing translational velocity. Accordingly, at higher translation velocities as are characteristic of flights under spatially less constrained conditions the spatial range within which environmental objects lead to significant intersaccadic response increments is extended to a few more meters (Ullrich et al., 2014a). From an ecological perspective it appears to be economical and efficient that the behaviorally relevant spatial range that is represented by motion detection systems scales with locomotion velocity: a fast moving animal can thus initiate an avoidance maneuver at a greater distance from an obstacle than when moving slowly.

We can conclude from this experimental evidence that during translational self-motion as is characteristic of the intersaccadic flight phases of flies and bees that even motion sensitive cells with relatively large receptive fields provide spatial information about the environment. Although it is still not clear to what extent this information is exploited for behavioral control (see below), its potential functional significance is underlined by the fact that the object-induced responses observed during intersaccadic intervals are further increased relative to the background activity of the cell as a consequence of motion adaptation (Liang et al., 2008, 2011, 2012; Ullrich et al., 2014b).

### Behavioral significance of motion-based spatial information

Fast flying animals, such as many insects, need to respond to environmental cues often already at some distance, for instance, when they have to evade a potential obstacle in their flight path or when using objects as landmarks in guiding them to a previously learnt goal location. Then optic flow is likely to be the most relevant cue to provide spatial information. Accordingly, motion cues have been implicated on the basis of many behavioral analyses to be decisive in controlling behavioral components of flying insects. Optic flow processing determines several aspects of the landing behavior (Wagner, 1982; Lehrer et al., 1988; Srinivasan et al., 1989, 2001; Kimmerle et al., 1996; Evangelista et al., 2010; van Breugel and Dickinson, 2012; Baird et al., 2013), and is used for flower distance estimation and tracking (Lehrer et al., 1988; Kern and Varjú, 1998). Insects also seem to exploit retinal motion in the context of collision avoidance (Tammero and Dickinson, 2002a,b; Reiser and Dickinson, 2003; Lindemann et al., 2008, 2012; Kern et al., 2012; van Breugel and Dickinson, 2012; Lindemann and Egelhaaf, 2013). Moreover, insects, such as bees and wasps, show a rich repertoire of visual navigation behavior employing motion cues on a wide range of spatial scales. When a large distance to a goal needs to be spanned, odometry, i.e., determining flown distances, based on optic flow cues is a central constituent of navigation mechanisms of bees (Srinivasan et al., 1997; Esch et al., 2001; Si et al., 2003; Tautz et al., 2004; Wolf, 2011; Eckles et al., 2012). However, even if the animal is already in the vicinity of its goal it can use spatial cues based on optic flow to find the goal (Zeil, 1993b; Lehrer and Collett, 1994; Dittmar et al., 2010, 2011), although also textural and other cues play an important role in local navigation (Collett et al., 2002, 2006; Zeil et al., 2009; Zeil, 2012). Bees even seem to orchestrate their flights in specific ways that facilitate gathering spatial information by intersaccadic movements with a strong sideways component (Lehrer, 1991; Zeil et al., 2009; Dittmar et al., 2010; Braun et al., 2012; Collett et al., 2013; Philippides et al., 2013; Riabinina et al., 2014; Boeddeker et al., submitted).

Turns, at least of flies and bees, are thought in most behavioral contexts including collision avoidance behavior to be accomplished in a saccadic fashion. Hence, understanding the mechanisms underlying collision avoidance means understanding by what visual input during an intersaccadic interval evasive saccades are elicited. There is consensus that intersaccadic optic flow plays a decisive role in controlling the direction and amplitude of saccades in this behavioral context. Despite discrepancies in detail, all proposed mechanisms of evoking saccades rely on extracting asymmetries between the optic flow patterns in front of the two eyes. Asymmetries may be due to the location of the expansion focus in front of one eye or to a difference between the overall optic flow in the visual fields of the two eyes (Tammero and Dickinson, 2002b; Lindemann et al., 2008, 2012; Mronz and Lehmann, 2008; Kern et al., 2012; Lindemann and Egelhaaf, 2013). Not all parts of the visual field have been concluded to be involved in saccade control of blowflies in the context of collision avoidance. The intersaccadic optic flow in the lateral parts of the visual field does not play a role in determining saccade direction (Kern et al., 2012). This feature appears to be functional as blowflies during intersaccades fly mainly forwards with only relatively small sideways components occurring mainly directly after saccades. These sideways components shift the pole of expansion of the flow field slightly towards frontolateral locations (Kern et al., 2012). In contrast, in *Drosophila*, which often hover and fly sideways (Ristroph et al., 2009), the optic flow and, thus, the spatial information sensed in lateral and even rear parts of the visual field has been concluded to be also involved in saccade control in the context of collision avoidance (Tammero and Dickinson, 2002b).

**Figure 7:**
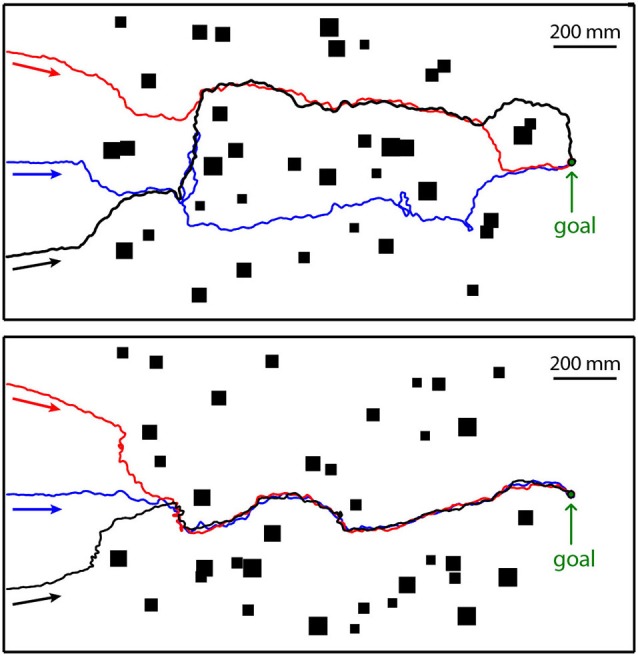
**Collision avoidance while heading for a goal**. The model insect starts at the left at three different positions (colored arrows) in two different cluttered environments (top and bottom diagrams). The goal is indicated at the right of the environment. The three resulting trajectories in each environment are given in red, blue and black. The objects as seen from above are indicated by black rectangles. The walls enclosing the environment are represented by thick black lines. The walls and the objects were covered with the same random texture. Direction of locomotion is indicated by arrows underneath trajectories (Data from Bertrand et al., submitted).

Nonetheless, systematic analyses based on models of LWCs with EMDs as their input revealed difficulties with regard to collision avoidance performance of a simulated insect arising from the contrast and texture dependence of the local motion detectors (Lindemann et al., 2008, 2012; Lindemann and Egelhaaf, 2013). The difficulties with these models can be reduced to some extent by implementing contrast normalization in the peripheral visual system (Babies et al., 2011). Recent modeling based on a somewhat different approach indicates an even more robust solution to the problem. Here, a spatial profile of the environment is determined along the horizontal extent of the visual field from local EMD-based motion measurements. The motion measurements are performed during short intersaccadic translatory flight segments. Although this spatial profile does not represent pure nearness information, but also the contours of nearby environmental structures (Figure 7), it allows determining a locomotion vector that points in the direction which makes a collision least likely and, thus, allows, under most circumstances, to avoid colliding with obstacles. This is even true when the objects are camouflaged by being covered with the same texture as the background of the environment (Bertrand et al., submitted). If the collision avoidance algorithm is combined with an overall goal direction, leading for example to a previously learnt food source or a nest, the model insect tends to move on quite similar trajectories to the goal through a heavily cluttered environment irrespective of the exact starting conditions by employing just the local motion-based collision avoidance mechanism, but no genuine route knowledge (Figure 7). It is interesting to note that these trajectories are reminiscent of routes of ants heading for their nest hole from different starting locations that are usually interpreted within the conceptual framework of navigation mechanisms (Wehner, 2003; Kohler and Wehner, 2005).

Whereas collision avoidance and landing are spatial tasks that must be solved by any flying insect, local navigation is relevant especially for particular insects, such as bees, wasps and ants, which care for their brood and, thus, have to return to their nest after foraging. Apart from finding without collisions a way towards the area where the goal may reside, motion information may be employed to determine the exact goal location by using the spatial configuration of objects, i.e., landmarks located in the vicinity of the goal (Lehrer, 1991; Zeil, 1993a,b; Lehrer and Collett, 1994; Collett and Zeil, 1996; Zeil et al., 2009; Dittmar et al., 2010, 2011; Braun et al., 2012; Collett et al., 2013; Philippides et al., 2013; Boeddeker et al., submitted). Motion information is especially relevant, if the landmarks are largely camouflaged by similar textural properties as those of the background (Dittmar et al., 2010). Information about the landmark constellation around the goal is memorized during elaborate learning flights: the animal flies characteristic sequences of ever increasing arcs while facing the area around the goal. During these learning flights, the animal is thought to gather relevant information about the spatial relationship of the goal and its surroundings. This information is subsequently used to relocate the goal when returning to it after an excursion (Collett et al., 2002, 2006; Zeil et al., 2009; Zeil, 2012). The mechanisms by which information about the landmark constellation is learnt and subsequently used to localize the goal are still controversial. However, optic flow information is likely to be required to detect texturally camouflaged landmarks and to derive spatial cues that are generated actively during the intersaccadic intervals of translational flight. Also textural cues characterizing the landmarks seem to be relevant for localizing the goal, since bees were found to adjust their flight movements in the vicinity of the landmarks according to the landmarks’ specific textural properties (Dittmar et al., 2010; Braun et al., 2012). It remains to be shown in future behavioral experiments and model analyses, whether the optic flow information and textural cues relevant for navigation performance can be accounted for on the basis of the joint velocity and texture dependence of biological movement detectors and of EMDs as their model equivalents. Alternatively, mechanisms may be required that process optic flow and environmental texture separately and combine both cues only at a later processing stage.

## Conclusions

The nearness of objects is reflected in the optic flow generated on the eyes during translational self-motion as is characteristic of the intersaccades of insect flight. In many behavioral contexts nearby objects are particularly relevant. Examples are obstacles that need to be evaded, landing sites, or landmarks that indicate the location of an inconspicuous goal. The main assumption of this review is that the behaviorally highly relevant spatial information can be gained without sophisticated computational mechanisms from the optic flow generated as a consequence of translational locomotion through the environment.

However, movement detectors as are widespread in biological systems and can be modeled by correlation-type EMDs do not represent veridically the velocity vectors of the optic flow, but rather also reflect textural information of the environment. This distinguishing feature has often been regarded as nothing but a nuisance of a simple motion detection mechanism. This opinion has been challenged recently by analyzing motion detectors with image flow as generated during translational movements through a wide range of cluttered natural environments. On this basis, the texture information has been suggested to be potentially of functional significance, because it basically reflects the contours of nearby objects. Contrast borders are thought for long to be the main carrier of functionally relevant information about objects in artificial and natural sceneries. This is evidenced by the well-established finding that contrast borders are enhanced by early visual processing in biological visual systems including that of primates (e.g., Marr, 1982; van Hateren and Ruderman, 1998; Simoncelli and Olshausen, 2001; Seriès et al., 2004; Girshick et al., 2011; Berens et al., 2012). One major function of this type of peripheral information processing is thought to be the enhancement of contrast borders at the expense of the overall brightness of the image, but also redundancy reduction in images. Independent of the particular conceptual framework, enhancing contrast borders is seen as advantageous with regard to representing visual environments.

The main conclusion of this paper is that the motion vision system of insects combines both nearness and contour information and preferentially represents contrast borders of nearby environmental structures and/or objects during translatory self-motion. It makes just use of the fact that in normal behavioral situations all this information is only required when an animal is moving. Then the motion vision system segregates, in a computationally parsimonious way, the environment into behaviorally relevant nearby objects and—at least in many behavioral contexts—less relevant distant structures. This characteristic matches—as we think—one major task of the motion detection system, to provide behaviorally relevant behavioral information about the environment, rather than only to extract the velocity of self-motion or the velocity of moving objects. Based on this conclusion, motion detection should not be conceptualized exclusively in the context of velocity representation, which is certainly important in many contexts, but also in the context of gathering behaviorally relevant information about the environment.

## Conflict of interest statement

The authors declare that the research was conducted in the absence of any commercial or financial relationships that could be construed as a potential conflict of interest.
